# Puberty in Context: Accounting for Psychosocial Experiences in the Association Between Pubertal Timing, Sex/Gender, and Adolescent Depressive Symptoms in Canadian Youth

**DOI:** 10.1155/da/5568871

**Published:** 2026-04-12

**Authors:** Annie Duchesne, Nicole White, Brielle C. Cooke, Caroline Sanders

**Affiliations:** ^1^ Department of Psychology, University of Northern British Columbia, Prince George, British Columbia, Canada, unbc.ca; ^2^ Department of Psychology, University of Quebec in Trois-Rivières, Trois-Rivières, Quebec, Canada; ^3^ School of Nursing, University of Northern British Columbia, Prince George, British Columbia, Canada, unbc.ca

**Keywords:** adolescent depressive symptoms, childhood adversity, postpubertal stress, pubertal timing, sex/gender differences

## Abstract

Literature suggests that early pubertal timing constitutes a risk factor for adolescent depression, especially in girls. However, adverse experiences preceding (e.g., childhood adversity), co‐occurring with (e.g., changing peer and parent relationships), and following puberty (e.g., delinquent behavior) have been associated with both pubertal timing and adolescent depression, and little is currently known about how these experiences may be differentially associated with the correspondence between pubertal timing and depression across sex/gender. Using Canadian data from the National Longitudinal Survey of Children and Youth (NLSCY), the present study investigated how sex/gender and relevant childhood, peri, and postpubertal psychosocial variables informed the relationship between pubertal timing and adolescent depressive symptoms. Preliminary models examining the relationship between pubertal timing and depressive symptoms in girls and boys suggested a linear association for both groups, facilitating a mixed‐sex/gender analysis supporting direct testing of sex/gender interactions. Data from 1400 Canadian youth (53% female; 96% Caucasian) showed that girls were more likely to report earlier pubertal development and higher rates of depressive symptoms at age 16–17. In a base model ignoring psychosocial context, we observed a significant interaction where early pubertal timing was associated with greater depressive symptoms in girls, with no pubertal timing effect apparent in boys. However, after accounting for psychosocial factors, adolescent depressive symptoms were best predicted by childhood emotional problems, peripubertal body dissatisfaction and perceived parental rejection irrespective of sex/gender, with additional sex/gender‐contingent effects of childhood family dysfunction and postpubertal delinquency. Moreover, pubertal timing did not explain significant variance in depressive symptoms in girls or boys after accounting for psychosocial context. These findings support the context‐contingency of sex/gender differences in the association between pubertal timing and adolescent depression. Conceptual and methodological ramifications of the need for an integrated biopsychosocial focus are discussed.

## 1. Introduction

The global prevalence of depression is approximately twice as high in women compared to men [1–3]. This sex/gender^1^ gap, which has grown wider in recent years [1, 4, 5], emerges in early adolescence, with global prevalence rates estimated at 32% for girls and 24% for boys [6]. Elevated prevalence of depression in girls becomes more pronounced as it persists into adulthood [3, 7], leading many to suggest that pubertal development constitutes a window of vulnerability for depression, especially in girls [8, 9].

Pubertal development encompasses numerous dimensions, including hormonal (e.g., activation of the hypothalamic–pituitary–gonadal axis), morphological (e.g., development of secondary sexual characteristics), physiological (e.g., menarche and nocturnal emission), and psychosocial changes (e.g., changes to peer groups). Although many of these dimensions of pubertal development have been associated with adolescent depression, the timing of pubertal changes has been most consistently associated with adolescent psychopathology across a wide range of conditions [10–13]. Pubertal timing is a peer‐relative measure, commonly computed by using self‐reported pubertal status measures (e.g., the Pubertal Development Scale) [14] to create age‐ and sex‐standardized pubertal timing scores that reflect whether development is earlier, on par, or later than that of peers ([15]; Supporting Information File 1 for different operationalizations of puberty).

Early pubertal timing has been associated with higher rates of adolescent depressive symptoms, and with adolescent psychopathology broadly, in a large‐scale meta‐analysis [11]. This small but significant pooled effect of early pubertal timing was observed irrespective of psychopathological domain (e.g., depressive symptoms vs. eating disorder), symptom severity (clinical vs. community samples) and sex/gender, although the ability to investigate sex/gender effects was limited by sampling bias in the available literature (80% of studies were conducted in female‐only samples). Physiologically, the association between early pubertal timing and adolescent depressive symptoms has been proposed to be mediated by a reduced cortico‐limbic connectivity impacting stress and emotion regulating processes [12, 16, 17]. Although more recent studies in large mixed‐sex/gender samples continue to report a significant association between early pubertal timing and adolescent depressive symptoms, findings are inconsistent with respect to sex/gender differences, with some studies observing early pubertal timing effects only in girls [18–20] while others report no apparent sex/gender‐related differences [21, 22]. These inconsistencies suggest that other variables could help to explain apparent sex/gender differences in the association between pubertal timing and adolescent depression.

Indeed, a growing literature suggests that whether early pubertal timing constitutes a risk factor for adolescent depression is contingent on the presence of stress‐related psychosocial experiences occurring before, during, and after pubertal development [8, 12, 23]. To date, only a handful of studies have investigated the role of psychosocial context in the sex/gender difference. Using Canadian data from the National Longitudinal Study of Children and Youth (NLSCY), Benoit et al. [24] observed that the association between pubertal timing and adolescent depression varied according to childhood and peripubertal psychosocial factors in both girls and boys. Specifically, elevated symptoms of depression at 16–17 were observed in girls who experienced both early pubertal timing and early dating (≤age 13), but not in girls reporting no early dating experiences. For boys, elevated depressive symptoms were observed only for those with early pubertal timing who also reported relationships with deviant peers. For both boys and girls, early pubertal timing also corresponded with elevated adolescent depressive symptoms only for those with high perceived parental rejection at age 14–15. Finally, a quadratic association between pubertal timing and depressive symptoms was also observed for boys only, where a combination of early or late pubertal timing and low levels of childhood emotional problems showed lower rates of depressive symptoms compared to “on‐time” developing boys. Importantly, this study conducted analyses separately by sex/gender, precluding clear conclusions regarding possible sex/gender‐related differences [25–27]. Nonetheless, these results point to a need to situate pubertal development in a psychosocial context, particularly accounting for childhood adverse emotional experiences and negative peripubertal peer and parental interactions, to better understand depression risk in adolescence across sex/gender.

Negative interpersonal experiences during pubertal development may be reasonably hypothesized to contribute to depression risk. One potentially central source of these experiences relates to body image and body dissatisfaction. Daniels et al. [28] synthesized literature on antecedents of self‐objectification in girls and reported that pubertal development in girls aged 10–12 is predictive of increased teasing and sexual harassment by peers, higher BMI, and greater feelings of body shame. Victimization, teasing, and sexual harassment by peers were in turn also associated with increased feelings of body shame, particularly in postmenarcheal girls [28], suggesting that body dissatisfaction in girls may be precipitated by the increased occurrence of negative psychosocial experiences during pubertal development. Comparable data regarding the association between pubertal timing and body dissatisfaction was largely unavailable for boys in this review, though other work suggests that pubertal timing is associated with experiences of body dissatisfaction in boys as well [29, 30]. Further, adolescent body dissatisfaction has been well documented as a strong predictor of depressive symptoms and as a potentially important mediator of sex/gender‐related differences in depressive symptoms [30–32]. Despite these associations between body dissatisfaction, pubertal timing, sex/gender, and depression, consideration of peripubertal body dissatisfaction as a form of negative psychosocial context remains underexplored.

Pubertal development has also been hypothesized to serve as a risk factor for the increased occurrence of postpubertal life stressors, which, in turn, may present a risk of depression in adolescence [33, 34]. This model was partially tested by Mendle et al. [35] in a female‐only sample drawn from the National Longitudinal Study of Adolescent and Adult Health to evaluate whether early puberty in girls precipitated stressful life experiences during adolescence that could help account for elevated depressive symptoms in adulthood. Postmenarcheal life events significantly predicted by age at menarche were conceptualized as events that may “snare” individuals and increase the risk of depression. Importantly, these life events were counted only if they first occurred postmenarcheally and included teenage criminal arrest, discontinued education, teenage pregnancy, and/or childbearing, experience of sexual assault by caregiver and/or nonfamily‐member, and experiences of physical assault by caregiver, nonfamily‐member, and/or intimate partner. Analysis revealed that the apparent association of adult depression with age at menarche was fully mediated by the occurrence of postmenarcheal life events to which early‐developing girls were disproportionately exposed, namely elevated rates of discontinued education and physical and sexual assault. Consequently, whether pubertal development itself serves as a risk factor for depression vs. as a risk factor for the occurrence of depressogenic life experiences is important to delineate further in the study of sex/gender differences in depression, as the incidence of numerous adverse life experiences is known to differ by sex/gender.

Finally, it is also important to consider that various childhood experiences are associated with the timing of pubertal development. Early experiences of threat [36–38], deprivation [39–41], unpredictable familial environment [42, 43], and cumulative trauma exposure [44, 45] have all been associated with variation in pubertal timing, although not consistently. Here it is proposed that negative childhood experiences would accelerate pubertal maturation and associated structural and functional brain development, particularly cortico‐limbic connectivity (reviewed in [36, 46]). Similarly, associations between early life adversities and pubertal timing have been shown to be moderated by sex/gender, although the presence and direction of such effects vary considerably between studies [40, 45, 47–51]. Negative childhood experiences have also been associated with variation in peri‐ and postpubertal interpersonal experiences such as body dissatisfaction and delinquency [52, 53]. Currently, most research interrogating sex/gender variation in the relation between early stress exposure, pubertal timing, and adolescent depressive symptoms fails to account for peri and postpubertal psychosocial experiences.

Considering the role of pubertal timing as a potential risk factor for adolescent depression and the importance of childhood, peri, and postpubertal psychosocial factors in the association between pubertal timing and depression [24, 35], the present study investigated the role of sex/gender in the relationship between pubertal timing and adolescent depressive symptoms when accounting for childhood, peri, and postpubertal experiences. A primary objective of this study is to critically interrogate whether early puberty itself is in fact a risk factor for depression or whether psychosocial experiences which covary with pubertal development better account for variance in adolescent depressive symptoms. If the latter is true, then studies in which pubertal development is examined without accounting for psychosocial risk factors may misidentify early puberty as a risk factor due to unexamined third‐variable effects. This is the central question of our work, motivated by converging meta‐analyses suggesting that overall effects of pubertal timing are small, with most studies overlooking psychosocial factors.

Based on available literature, we predicted that the association between pubertal timing and adolescent depressive symptoms would be contingent upon psychosocial context and that any effects of psychosocial context may vary in a sex/gender‐related manner.

## 2. Methods

### 2.1. Participants

The National Longitudinal Survey of Children and Youth (NLSCY) was conducted by Statistics Canada [54] to capture social, emotional, and behavioral development and wellbeing over time in a representative sample of Canadian children and youth across 10 Canadian provinces. The survey includes both self‐report measures completed by children/youth as well as measures completed by the person most knowledgeable about the child (PMK) over 18 years of age in the household, typically a parent. The first cohort (cycle 1) was collected in 1994 and included children aged 0 to 11 years who were followed longitudinally across eight data collection cycles on a biannual schedule until the final cycle was collected in 2008. Data collection cycles after cycle 1 supplemented the original sample with the addition of cohorts comprising children aged 0 to 1 at the end of the year prior to that collection cycle. The present analyses focus on a subsample of participants who were aged 8 to 11 years old at cycle 1. Further details regarding the identification of the analytic sample and handling of missing data within and across survey cycles are available in Supporting Information File 1.

### 2.2. Measures

#### 2.2.1. Demographics

Several demographic variables were coded, including geographical location (rural/urban designation), PMK gender, number of people residing in the household, highest education level of PMK at cycle 1, age, gender (options: male or female), immigration status, country of birth, ethnicity, and socioeconomic status at cycle 1 (indexed via a continuous composite measure capturing income, education, and occupational prestige [54].

#### 2.2.2. Pubertal Status

Pubertal status (prepubertal vs. postpubertal) was coded at each of cycles 1–5 using responses from the Pubertal Development Scale (PDS; [14]). As puberty is not a binary process with simple categorical boundaries, for the purpose of the present study, “postpubertal” can be taken to mean, broadly, “not prepubertal,” or in other words, capturing youth at any stage of pubertal development when puberty is underway. This binary variable was used (1) to create an age‐anchored variable indexing the timing of pubertal development (i.e., early puberty defined as endorsing postpubertal status ≤ age 11; [50]), and (2) to ensure that postpubertal experiences captured in analysis were the first occurrence reported by the participant (i.e., if participants reported an experience both pre and postpubertally, the postpubertal experience was coded as “not occurred”) following the procedure of Mendle et al. [15]. The PDS is a five‐item scale evaluating the appearance of secondary sex characteristics, including body hair growth (girls and boys), breast development and first menarche (girls), and voice change and facial hair growth (boys). Four items are rated on a four‐point ordinal scale with options: not yet started, barely started, definitely underway, and seems complete. The item regarding the first occurrence of menarche has responses yes and no. Girls were coded as postpubertal if they answered “definitely underway” or “seems complete” to both body hair growth and breast development *or* if they answered yes to the first occurrence of menarche, and coded as prepubertal otherwise. Boys were coded as postpubertal if they answered “definitely underway” or “seems complete” to items assessing body hair growth and voice change, as these changes typically occur prior to the appearance of facial hair [15].

#### 2.2.3. Pubertal Timing and Early Puberty

Following the procedures of Benoit et al. [24], we used data from cycles 2 and 3 to capture a point estimate of pubertal timing at ages 12–13, computing age‐ and sex‐standardized scores on the PDS to produce a continuous measure of pubertal timing where higher scores reflect earlier pubertal timing relative to same‐age, same‐sex peers. Using pubertal status across cycles, participants were also categorized as having early puberty if they were coded as postpubertal at or prior to age 10–11 (cycles 1 and 2) or as on‐time/late (i.e., not early) puberty if they were coded as prepubertal at ages 10–11. Peer‐relative measures introduce an element of social comparison to the operationalization of pubertal development, while anchoring the timing of physical development to age places more focus on individual developmental milestones (see Supporting Information File 1 for definitions). We examined both constructs to assess whether the operationalization of early pubertal development as peer‐relative vs. anchored to children’s age is associated with meaningful differences in observed outcomes, as suggested by Mendle et al. [15].

#### 2.2.4. Depressive Symptoms

Depressive symptoms were assessed by self‐report using the 12‐item Centre for Epidemiological Studies Depression scale (CES‐D; [55]) for all participants (i.e., children and PMK). For the present study, we considered adolescent depressive symptoms captured at ages 16–17 using data drawn from cycles 4 and 5. Descriptive data for depressive symptoms in adulthood at cycle 8 are also summarized below.

#### 2.2.5. Childhood Stressors

A total of 16 childhood stressors were categorized into three dimensions: loss, instability, and adversity [56]. Items pertaining to childhood life stressors were coded for children aged 10–11 using PMK‐reported data from cycles 1 and 2. Each item was coded as a 1 if it occurred at or prior to age 11, and 0 otherwise. Individual items were then grouped into composite stressor domains. Loss included reporting a parent or family member’s death; instability encompassed father absence, moving house, experiences of stay in foster care, changes in household membership, divorce, or other separation of parents. Finally, adversity/health stressors encompassed experiences involving harm, threat of harm, and trauma and included reports of abuse or fear of abuse, conflict between parents, witnessing violence in the home, alcohol use and/or mental health issues in the family, injury or illness of a family member, “other experiences of trauma,” hospital stay, and injury or illness of the child.

#### 2.2.6. Childhood Emotional, Behavioral, or Family Problems

Following the work of Benoit et al. [24] we coded several variables indexed by the PMK report for children ages 10–11 at cycles 1 and 2, including emotional problems (e.g., sadness and worry), conduct problems, PMK depression (CES‐D) scores, and family dysfunction (Supporting Information File 1 for additional information on measures). All measures were continuous.

#### 2.2.7. Peripubertal Risk Factors

Perceived parental rejection at ages 14–15 (cycles 4 and 5) was identified by Benoit et al. [24] to comprise a significant predictor of adolescent depressive symptoms. We also examined other items identified by Benoit et al. [24] including perceived peer popularity and early dating; however, due to the addition of postpubertal life events and prepubertal stressor variables, we did not analyze these items due to substantial missing data that would too severely limit the analytic sample size.

Peripubertal body dissatisfaction was captured at ages 14–15 (cycles 4 and 5) using a single self‐report item capturing participants’ agreement with the statement “I have a good body” with Likert response options collapsed into a binary agree/disagree outcome.

#### 2.2.8. Postpubertal Life Events

Identification of items for coding postpubertal life events was guided by Mendle et al. [35], and included indices of discontinued education, teenage pregnancy, and various items related to participation in delinquent behavior (e.g., theft from family members or others, vandalism or other destruction of property, perpetrating physical or sexual assault, and so on; Supporting Information File 1). Discontinued education and teenage pregnancy did not have adequate numbers reported to include in subsequent analyses and are not discussed further here. Association with deviant peers was coded following Benoit et al. [24] as a binary variable captured at ages 14–15. Fifteen items pertaining to postpubertal delinquency were evaluated at all available cycles and combined into composite variables for analysis (Supporting Information File 1). First, we coded the occurrence of each item (yes/no) at every cycle, followed by evaluating at each available cycle whether the item was reported pre or postpubertally. If items were reported prepubertally or not reported, they were coded 0; occurrence was coded as 1 only if the first reported occurrence was postpubertal, following the procedure of Mendle et al. [35]. Delinquency items were combined into two composite variables reflecting covert and overt delinquent behaviors [57, 58]; Supporting Information File 1.

### 2.3. Analysis

Our analysis plan generally follows that of Mendle et al. [35] with some adaptations to accommodate a mixed‐sex sample and interaction testing. Analysis proceeded in three stages, all using data‐driven model‐fitting, and was conducted using R [59] and the survey package [60]. Continuous variables were mean‐centered using survey‐weighted means prior to analysis. Post hoc testing for categorical variables was conducted by reanchoring regression models to assess effects at relevant design cells, while testing of continuous variable effects was conducted using simple slopes at ±1SD from the mean per procedures recommended by Aiken and West [61].

Descriptive statistics for cycle 1 demographics comparing participants who completed all cycles of data collection with those lost to attrition were computed using cycle 1 cross‐sectional weights. All regression analyses conducted subsequently were ordinary least squares regression models weighted using cycle 8 longitudinal nonfunnel weights adjusted for attrition. All weights were provided by Statistics Canada [54]. Weighting is strongly recommended to account for Statistics Canada’s use of clustered sampling procedures and attrition (Supporting Information File 1).

We first estimated a model predicting pubertal timing from all identified childhood stressors together with variables captured at or prior to age 11 (cycle 1), including PMK depression scores, childhood emotional problems, conduct problems, and family dysfunction. Next, each term in this model was sequentially tested in two–way interaction with sex/gender to determine whether any predictor demonstrated a sex/gender‐contingent association with pubertal timing. Log‐likelihood ratio (LLRT) comparisons were used to evaluate changes to overall model fit. A nonsignificant LLRT favours selection of the simpler of two models. Next, a model was computed adding all interaction terms found to improve model fit. Finally, the model was back‐fitted, again using LLRT to compare models after removing each term in order of smallest *t*‐value, until no further terms could be removed without reducing the explanatory power of the model.

To examine the relationship between sex/gender, pubertal timing, and depressive symptoms at age 16–17, we computed preliminary models to determine whether the use of pooled or sex‐disaggregated analysis was better supported by the data. We estimated a preliminary model predicting depressive symptoms from pubertal timing, comparing a linear‐only model and a model with both linear and quadratic terms, according to previous work [24, 62, 63]. To test for the presence of sex/gender‐contingent differences in the fit of linear versus quadratic terms, we also computed comparison models predicting depressive symptoms from sex/gender × pubertal timing (linear) against a model with both sex/gender × pubertal timing (linear) and sex/gender × pubertal timing (quadratic). No statistical support was found for the inclusion of a quadratic term for pubertal timing overall or in a sex/gender‐contingent manner. As such, the data in the present sample statistically support a mixed‐sex analysis using only a linear term for standardized pubertal timing.

Subsequently, we considered the linear sex/gender ^∗^pubertal timing model to serve as our base model for predicting adolescent depressive symptoms. All other identified variables were added to this model (i.e., childhood, peripubertal, and postpubertal variables). Next, we sequentially tested each model term in interaction with pubertal timing, using LLRT comparisons to justify retaining interaction terms. Then, similarly, we tested each predictor in interaction with sex/gender. Interaction terms were found to improve model fit were included together in an interaction model. Three–way interaction terms were only tested if a single factor was involved in more than one two–way interaction. Finally, the resulting model was backfitted removing terms in order of smallest t‐value until no further terms could be removed without reducing fit. We present models for pubertal timing and some preliminary findings related to categorical pubertal development status (early/not early).

Finally, of the six available peri and postpubertal variables included in the analysis, we conducted follow‐up analyses on three that were associated with adolescent depressive symptoms (covert delinquency, body dissatisfaction, and perceived parental rejection) to explore whether childhood factors, sex/gender, and pubertal timing accounted for variance in these outcomes. Analyses were conducted similarly to those for predicting depressive symptoms, with the exception that we did not conduct further testing of quadratic terms for pubertal timing, nor did we include peri and postpubertal predictor variables as predictors of peri and postpubertal variables due to the contemporaneous nature of these factors. Predictors tested included cycle 1 SES, all childhood variables captured at or prior to age 11, pubertal timing, and sex/gender. Each term was tested in interaction with both pubertal timing and sex/gender.

## 3. Results

### 3.1. Descriptive Statistics

Biases were apparent in survey attrition and completion rates. Comprehensive information regarding demographics according to participant attrition and missing data resulting in participant exclusion is available in Supporting Information File 1 and Tables S1 and S2.

### 3.2. Demographics of Sample

The analytic sample (unweighted *n* = 1400) is described according to sex/gender in Table 1. This sample is comparable in size to the analytic sample examined by Benoit et al. [24], also using the NLSCY. We initially planned to include all psychosocial variables identified by Benoit et al. [24] as relevant to adolescent depression. However, with the additional inclusion of postpubertal variables, there were substantial missing data on some items examined by Benoit et al. [24] (e.g., early dating) that would have restricted the analytic sample size too severely. Overall, variables included in the present work and in the work of Benoit et al. [24] were descriptively similar (e.g., cycle 1 SES; perceived parental rejection).

**Table 1:** Descriptive summary by sex.

Variable	Girls	Boys	*p*‐Value
	Weighted *N* = 262,371	Weighted *N* = 224,242	
Cycle 1 SES			
Mean (SE)	0.07 (0.06)	0.21 (0.06)	*p* = 0.072
95% CI	[−0.04, 0.18]	[0.10, 0.32]	
Cycle 1 PMK highest education			
No postsecondary education	32.6	28.2	*p* = 0.302
Postsecondary education	67.4	71.8	
Timing of pubertal development^b^			
Not early (≥age 11)	79.0	93.4	**p < 0.0001**
Early (≤age 11)	21.0	6.6	
Childhood loss (child age ≤10–11)			
No loss	74.0	85.0	**p = 0.006**
Loss reported	26.0	15.0	
Childhood instability (child age ≤10–11)			
No instability	82.8	87.1	*p* = 0.145
Instability reported	17.2	12.9	
Childhood adversity (child age ≤10–11)			
No adversity	64.9	64.1	*p* = 0.859
Adversity reported	35.1	35.9	
Childhood health stressors (child age ≤10–11)			
No health stressors	95.0	96.2	*p* = 0.467
Health stressors reported	5.0	3.8	
PMK depression score (child age 10–11)			
Mean (SE)	4.3 (0.4)	3.7 (0.3)	*p* = 0.174
95% CI	[3.6, 5.1]	[3.1, 4.3]	
Childhood conduct problems (age 10–11)			
Mean (SE)	0.9 (0.1)	1.2 (0.1)	*p* = 0.087
95% CI	[0.8, 1.1]	[1.0, 1.4]	
Childhood family dysfunction (age 10–11)			
Mean (SE)	7.6 (0.3)	7.7 (0.3)	*p* = 0.907
95% CI	[7.1, 8.2]	[7.1, 8.3]	
Childhood emotional problems (age 10–11)			
Mean (SE)	2.9 (0.2)	2.6 (0.2)	*p* = 0.317
95% CI	[2.5, 3.3]	[2.3, 3.0]	
Peripubertal perceived parental rejection (age 14–15)			
Mean (SE)	10.4 (0.3)	10.9 (0.4)	*p* = 0.309
95% CI	[9.8, 11.1]	[10.2, 11.6]	
Peripubertal body dissatisfaction (age 14–15)			
Not dissatisfied	60.6	60.6	*p* = 0.999
Dissatisfied	39.4	39.4	
Peripubertal deviant peer affiliation (age 14–15)			
No	69.0	63.3	*p* = 0.197
Yes	31.0	36.7	
Postpubertal covert deliquency^a^			
None	38.5	38.9	*p* = 0.935
Any covert delinquency	61.5	61.1	
Postpubertal overt delinquency^a^			
None	63.1	68.6	*p* = 0.197
Any overt delinquency	36.9	31.4	
Postpubertal assault^a^			
None	88.7	81.3	**p = 0.020**
Assaulted	11.3	18.7	
Adolescent depression score			
Mean (SE)	9.5 (0.4)	7.7 (0.4)	**p = 0.002**
95% CI	[8.7, 10.2]	[7.0, 8.5]	
Adolescent probable depression (CES‐D ≥ 12)			
Not depressed	70.3	78.2	*p* = 0.061
Somewhat depressed or worse	29.7	21.8	
Adult depression score (cycle 8)			
Mean (SE)	4.4 (0.3)	3.7 (0.3)	*p* = 0.086
95% CI	[3.8, 5.0]	[3.1, 4.2]	
Adult probable depression (CES‐D≥12 @ Cycle 8)			
Not depressed	89.7	95.5	**p = 0.020**
Somewhat depressed or worse	10.3	4.5	

*Note:* All categorical data are represented as survey‐weighted % of sample reporting. *p*‐Values derived from survey‐weighted chi‐square test for count data and tt‐test or ANOVA as relevant for continuous variables. PMK = person most knowledgeable about the child for caregiver‐reported variables. Bold values indicate statistical significance at *p* < 0.05.

^a^Postpubertal variables were coded to reflect the first occurrence of an experience postpuberty (i.e., participants with both pre and postpubertal reporting were coded as not occurred).

^b^Continuous pubertal timing scores were standardized by age and gender in the analytic sample to have M (SD) = 0 (1) for each cohort.

As documented in previous work [15], girls were more likely to report experiencing early puberty, with 21% of girls endorsing items on the PDS resulting in a code of postpubertal status at or prior to age 11 compared with just 6.6% of boys. Mean CES‐D scores in adolescence were significantly higher in girls, though the prevalence of probable depression (CES‐D ≥ 12) was only marginally elevated (*p* = 0.061). Interestingly, by adulthood (cycle 8), mean CES‐D scores did not differ by sex/gender, while the proportion of the sample scoring above the cut‐off was significantly higher in women (10.3% vs., 4.5%, *p* = 0.020). The overall prevalence of probable depression in the final sample at cycle 8 was 7.6%, slightly higher than the global prevalence rate of 5% in adults [64]. Adolescent probable depression was 26.1% overall, consistent with previous work indicating an overall higher prevalence of depression for this age range compared to adulthood [65–67]. The only other significant bivariate differences observed by sex/gender included a higher frequency of childhood loss reported by girls (26% vs. 15%, *p* = 0.006) and a higher frequency of experiences of postpubertal physical assault reported by boys (18.7% vs. 11.3%, *p* = 0.020). Note that this measure of assault does not index experiences of sexual violence.

### 3.3. Childhood Context as a Predictor of Pubertal Timing

#### 3.3.1. Standardized Pubertal Timing Scores

The final fitted model indicated that the only significant predictor of pubertal timing was cycle 1 SES (*b* = −0.194, SE = 0.069, *p* = 0.005; see Supporting Information File 2 for regression tables).

#### 3.3.2. Categorical Early Puberty (≤Age 11)

The final model demonstrated that the only significant predictor of the likelihood of experiencing early puberty based on physical development was sex/gender (*b* = −1.320, SE = 0.328, *p* < 0.0001).

### 3.4. Sex/Gender, Pubertal Timing, and Depressive Symptoms in Context

The base model predicting depressive symptoms from only sex/gender × pubertal timing showed a significant interaction (*t* = −2.27, *p* = 0.024; Table 2). In this model, there was a significant difference in CES‐D scores by sex/gender for those with on‐time development (*M*_female_ = 9.5 vs. *M*_male_ = 7.7; *t* = −3.28, *p* = 0.001). This difference was more pronounced for those with earlier pubertal timing (*M*_female_ = 10.4 vs. *M*_male_ = 7.4) and less pronounced for youth with later pubertal timing (*M*_female_ = 8.5 vs. *M*_male_ = 8.0).

**Table 2:** Base model: sex/gender × pubertal timing predicting depressive symptoms at age 16–17.

Coefficients	Estimate	Std. error	*t*‐Value	*p*‐Value	Lower CI	Upper CI
(Intercept)	9.479	0.396	23.932	< 0.0001	8.702	10.256
Pubertal timing	0.937	0.348	2.695	0.007	0.255	1.619
Participant gender (ref. female)	−1.751	0.534	−3.276	0.001	−2.800	−0.702
Pubertal timing × participant gender	−1.246	0.549	−2.268	0.024	−2.324	−0.168

*Note:* if no reference level is noted, variable is continuous and survey‐weighted grand mean has been used to center variable for analysis.

However, in the final model (Table 3), the sex/gender × pubertal timing interaction became nonsignificant after accounting for psychosocial context. The pubertal timing term was not retained in the model, and there was no main effect of sex/gender on depressive symptoms. Rather, depressive symptoms were significantly associated with childhood emotional problems, peripubertal perceived parental rejection, and peripubertal body dissatisfaction, higher scores on all of which were associated with significantly higher CES‐D scores. Two interactions were also significant: sex/gender × postpubertal covert delinquency, and sex/gender × childhood family functioning.

**Table 3:** Final back‐fitted model predicting adolescent depression (CES‐D) scores from pubertal timing, sex/gender, and identified covariates and moderators.

Coefficients	Estimate	Std. error	*t*‐Value	*p*‐Value	Lower CI	Upper CI
(Intercept)	6.903	0.462	14.955	<0.0001	5.997	7.808
Childhood emotional problems	0.313	0.113	2.775	0.006	0.092	0.534
Childhood family dysfunction	−0.070	0.068	−1.018	0.309	−0.204	0.065
Participant gender (ref. female)	0.056	0.653	0.085	0.932	−1.226	1.338
Peripubertal perceived parental rejection	0.264	0.055	4.820	0.000	0.157	0.372
Peripubertal body dissatisfaction (ref. satisfied)	1.585	0.506	3.135	0.002	0.593	2.578
Post‐pubertal covert delinquency (ref. none)	3.037	0.587	5.175	<0.0001	1.886	4.189
Participant gender × childhood family dysfunction	0.254	0.097	2.633	0.009	0.065	0.444
Participant gender × postpubertal covert delinquency	−2.884	0.943	−3.057	0.002	−4.735	−1.032

*Note*: if no reference level is noted, variable is continuous and survey‐weighted grand mean has been used to center variable for analysis.

No effect of childhood family dysfunction was observed in relation to depressive symptoms in girls, while boys with higher childhood family dysfunction scored significantly higher on the CES‐D (*b* = ‐0.254, *SE* = 0.097, *t =* 2.63, *p* = 0.009; Figure 1a). In contrast, regarding postpubertal covert delinquency, we observed no difference in mean depressive symptoms as a function of self‐reported participation in covert delinquent behavior in boys, while girls who reported covert delinquency scored significantly higher on the CES‐D compared to girls with no reported covert delinquency (*b* = 3.037, SE = 0.587, *t* = −3.06, *p* < 0.0001; Figure 1b).

Figure 1Mean CES‐D scores at age 16–17. (a) Sex/gender × childhood family dysfunction and (b) sex/gender × postpubertal covert delinquency.  ^∗^ *p* < 0.05.

**Figure:**
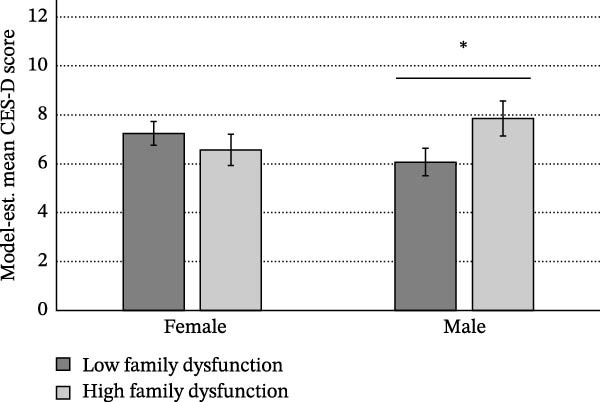
(a)

**Figure:**
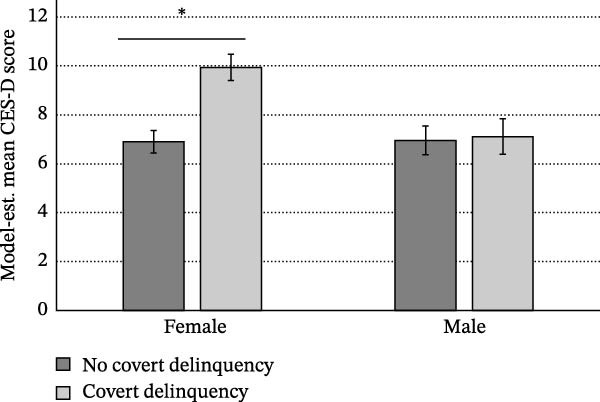
(b)

#### 3.4.1. Comparisons With Categorical Pubertal Development

We also conducted the same set of analyses replacing standardized continuous pubertal timing scores with our categorical designation of early (≤age 11) vs. not‐early (i.e., on‐time or late) puberty. This model was identical to the final model for standardized pubertal timing (see Supporting Information File 2 for regression tables).

### 3.5. Childhood Psychosocial Factors Predicting Peri and Postpubertal Context

All regression tables are available in Supporting Information File 2.

#### 3.5.1. Peripubertal Body Dissatisfaction

Using standardized pubertal timing scores, the final model predicting the likelihood of reporting body dissatisfaction (yes/no) at age 14–15 included significant effects of emotional problems at age 10–11, experiences of childhood loss and adversity/health stressors, and the only significant sex/gender × pubertal timing interaction observed in any of the final models in the present study (*b* = 0.435, SE = 0.217, *t* = −2.01, *p* = 0.045). Examination of simple slopes for the sex/gender × pubertal timing interaction confirmed a statistically weak interaction, as all post hoc comparisons were marginal or nonsignificant. Higher emotional problems at age 10–11 were associated with a higher likelihood of reporting body dissatisfaction (*b* = 0.123, SE = 0.038, *t* = 3.28, *p* = 0.001), while experiences of childhood loss (*b* = ‐0.700, SE = 0.242, *t* = −2.89, *p* = 0.004) and adversity/health stressors (*b* = ‐0.601, SE = 0.192, *t* = −3.13, *p* = 0.002) were each associated with a significantly lower likelihood of body dissatisfaction.

**Figure 2:**
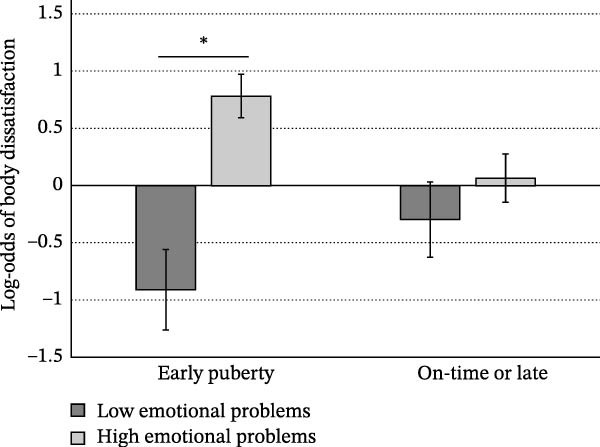
Childhood emotional problems × pubertal development predicting the likelihood of body dissatisfaction at age 14–15. Early puberty is defined as pubertal onset ≤age 11 (Supporting Information File 1).  ^∗^ *p* < 0.05.

Using categorical pubertal development, similar effects of early childhood loss and threat/adversity stressors were observed (both *p*s <0.003). In this model, no sex/gender × pubertal development interaction was observed, and a cycle 1 SES × sex/gender interaction was significant (*b* = 0.546, SE = 0.248, *t* = 2.20, *p* = 0.003). Girls with low cycle 1 SES were significantly more likely to report body dissatisfaction than girls with high cycle 1 SES (*p* = 0.043) while no significant effect of SES was observed for boys. Finally, a significant interaction was observed for pubertal development  ^∗^childhood emotional problems (*b* = 0.258, SE = 0.097, *t* = 2.65, *p* = 0.008; Figure 2). Post hoc tests revealed that the likelihood of reporting body dissatisfaction was significantly elevated in children with early physical development and high reported emotional problems. No effect of emotional problems was observed for children with on‐time or late physical development.

#### 3.5.2. Peripubertal Perceived Parental Rejection

**Figure 3:**
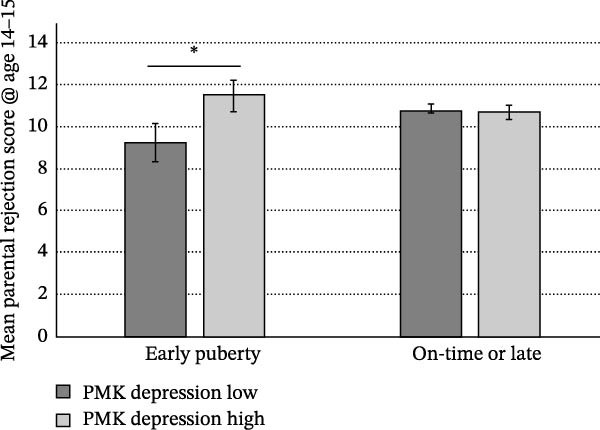
Pubertal development × PMK depression at age 10–11 predicting perceived parental rejection at age 14–15. Early puberty is defined as pubertal onset ≤ age 11 (Supporting Information File 1).  ^∗^ *p* < 0.05.

Only family dysfunction at age 10–11 explained significant variance in perceived parental rejection (*b* = 0.179, *SE* = 0.0439, *t* = 4.08, *p* < 0.0001) such that higher family dysfunction was associated with higher perceived parental rejection. No other terms were retained in this final model. However, in our second model considering categorical early pubertal development (≤ age 11), we also observed a significant interaction between pubertal development and PMK depression scores (*b =* −0.220, *SE* = 0.109, *t* = −2.03, *p* = 0.043; Figure 3). Those who experienced early development of secondary sex characteristics had lower levels of perceived parental rejection if PMK depressive symptoms were also low.

#### 3.5.3. Postpubertal Covert Delinquency

**Figure 4:**
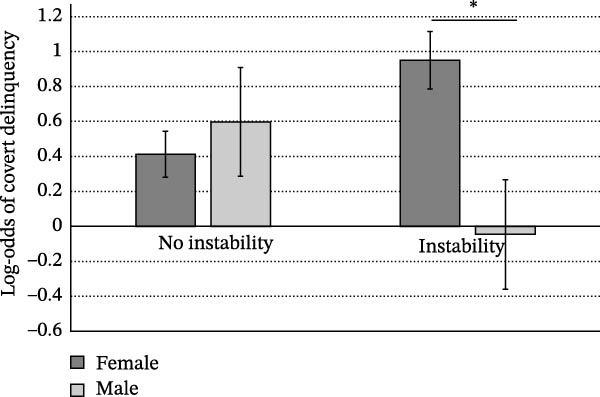
Sex/gender × childhood instability predicting the likelihood of postpubertal covert delinquency.  ^∗^ *p* < 0.05.

Self‐reported participation (yes/no) in covert delinquent activities was significantly predicted by higher PMK depression scores at age 10–11 and a sex/gender × childhood instability interaction for both designations of pubertal development. Girls with experiences of childhood instability were significantly more likely to report postpubertal covert delinquent behaviors than boys, while no sex/gender effect was apparent for children with no experiences of instability (Figure 4). Early pubertal timing (standardized) was also significantly associated with higher frequency of self‐reported covert delinquency (*b =* 0.292, SE = 0.094, *t* = 3.12, *p* = 0.002) similarly for girls and boys, while in a second, otherwise identical model, early physical pubertal development did not predict covert delinquency.

## 4. Discussion

The present study used data from the NLSCY to (1) test whether sex/gender moderated associations between two measures of pubertal development and self‐reported depressive symptoms at age 16–17, and (2) assess whether these interactions changed when effects of sex/gender were tested alongside children’s psychosocial context, including childhood, peri, and postpubertal psychosocial variables associated in previous work with pubertal development, sex/gender, and/or adolescent depressive symptoms. When tested in isolation, sex/gender moderated the association between pubertal timing and adolescent depressive symptoms. However, no such moderating effect was observed when pubertal development was operationalized as early physical maturation. Neither measure of pubertal development was retained in the final model predicting depressive symptoms in context, and sex/gender effects were only observed as a moderator of some psychosocial factors. These findings suggest that psychosocial experiences that covary with pubertal development are better predictors of adolescent depressive symptoms than pubertal development and binary sex/gender. The present findings contribute to highlighting the importance of developing a contextualized understanding of psychosocial experiences preceding, during, and following pubertal development in relation to adolescent depressive symptoms.

### 4.1. Adolescent Depressive Symptoms in Context

Early pubertal development has been found to predict increased depressive symptoms in later adolescence, an effect often reported to be more prevalent in girls compared to boys [9]. In our unadjusted base model, girls with earlier pubertal timing reported greater depressive symptoms than girls with average or later pubertal timing, with no effect of pubertal timing observed for boys, in alignment with previous work [9, 18, 19]. Moreover, mean depressive symptom scores were higher in girls than in boys irrespective of pubertal timing, with this difference being smallest for children with later pubertal timing. However, after accounting for relevant psychosocial experiences, this interaction became nonsignificant, pubertal development no longer contributed to explaining significant variance in depressive symptoms, and no independent effects of sex/gender on depressive symptoms were observed. This finding aligns with a growing literature supporting the mediating effects of psychosocial experiences [35, 68–71] on the relation between pubertal development and adolescent depression. Articulating pubertal development as a biosocial phenomenon, this literature highlights how variation in the timing of pubertal development is often associated with increased likelihood of psychosocial experiences that are in turn associated with depressive symptoms [35]. Our findings additionally suggest that psychosocial context fully accounts for the moderating role of sex/gender in the previously observed relationship between pubertal development and depression. Higher rates of depressive symptoms at age 16–17 were best predicted by body dissatisfaction at age 14–15, higher perceived parental rejection at age 14–15, and higher levels of emotional problems at age 10–11 irrespective of sex/gender. Finally, sex/gender demonstrated moderating effects on family dysfunction at age 10–11 and postpubertal covert delinquency in relation to depressive symptoms (Figure 1).

#### 4.1.1. Childhood Emotional Problems and Peripubertal Perceived Parental Rejection

In alignment with Benoit et al. [24], we observed that PMK‐reported childhood emotional problems and peripubertal perceived parental rejection were significantly associated with adolescent depressive symptoms in both boys and girls. However, in contrast to their sex‐segregated analyses demonstrating a moderating effect of pubertal timing on these variables in both groups, we did not observe interactions with pubertal timing when examining girls and boys together. The lack of replicating interaction findings may have occurred for several reasons. First, our study used a different analytical approach to exploring sex/gender differences (i.e., mixed‐sex vs. sex‐segregated analysis). Additionally, the analytical sample differed between the two studies due to variable selection constraints and the inclusion of variables such as body dissatisfaction, which were not evaluated by Benoit et al. [24] Nonetheless, converging evidence from these studies and others [35, 48, 71–73] highlights the importance of accounting for these childhood and peripubertal experiences in understanding adolescent depressive symptoms.

Childhood emotional problems at age 10–11 predicted depressive symptoms at age 16–17 irrespective of sex/gender and pubertal development. These findings align with those of recent population‐based longitudinal studies, which suggest stability in internalizing symptoms from late childhood to adolescence [74, 75]. Katsantonis [75] used latent growth curve modeling to explore continuous and discontinuous aspects of internalizing symptoms across development in children ages 4–16 and statistically identified a “transition point” for internalizing symptoms from late childhood to early adolescence. Specifically, children’s internalizing symptoms prior to age 10 showed a decreasing trend across development before beginning to increase again, after which symptoms were stable after age 10 until demonstrating another increase between ages 14–16. Consequently, Katsantonis [75] suggested that this transition point at the onset of early adolescence may be a critical period for the development of internalizing symptoms that is invariant to potential protective experiences in earlier childhood.

Higher levels of perceived parental rejection at age 14–15 also predicted more depressive symptoms at age 16–17 similarly for girls and boys. Interestingly, in our sample, children who physically matured early reported more perceived parental rejection at age 14–15 if their PMK had high levels of depressive symptoms when children were age 10–11 (Figure 3). Previous work indicates that pubertal development is associated with increased emotional distance in parent–child relationships regardless of children’s chronological age [76], an effect that could be amplified under conditions of parental depression. This effect was not apparent when examining age‐ and sex‐standardized pubertal timing, suggesting that early physical maturation at or before age 10–11 occurring within a negative parental environment may amplify experiences of parental rejection during later stages of pubertal development.

Our findings align with literature generally suggesting that perceived parental rejection is negatively associated with adolescent mental health [77–80], though sex/gender effects vary by study. Yu et al. [80] found evidence for a bidirectional association between parental rejection and adolescent depression in recent longitudinal work conducted with a large sample of youth living in China. Specifically, perceived maternal rejection did not differ by sex/gender and was associated with higher rates of adolescent depressive symptoms, which in turn were associated with increased reporting of paternal and maternal rejection at the second data collection time point 6 months later.

Using data from the NLSCY, Tisseyre et al. [79] conducted latent class analysis to identify vulnerability subtypes among a suite of 18 factors commonly associated with depression and reported a five‐class solution in which the classes they deemed “highest vulnerability” were characterized by high probability of perceived parental rejection at age 14–15, low parental nurturance, and low self‐esteem, among other factors. A majority (72%) of participants falling into the highest vulnerability class were girls, a sex/gender difference that was significant according to the chi‐square test. In turn, being classed as “high vulnerability” was strongly associated with depressive symptoms at age 16–17, an effect that did not interact with sex/gender [79]. In this study, self‐esteem was suggested to be a possible protective factor against high vulnerability to adolescent depression.

#### 4.1.2. Peripubertal Body Dissatisfaction

Negative body image and body dissatisfaction have been previously shown to predict adolescent depressive symptoms in both boys and girls. In some instances, the inclusion of body dissatisfaction in models of adolescent depression fully mediates apparent sex/gender differences in symptoms [32], while serving in other studies as only a partial mediator [30, 31]. Examining symptoms of depression in relation to sex/gender and body image, Mullarkey et al. [81] conducted a network analysis in an ethnically and socioeconomically representative sample of ~1400 youth living in the United‐States and found that self‐hatred was the most central (i.e., most potentially influential) symptom of adolescent depression, and that this symptom was strongly associated with negative body image. Further investigation of sex/gender‐stratified networks demonstrated a similar symptom network structure by sex/gender despite higher mean scores reported by girls on numerous symptoms, suggesting that the nature of the association between poor body image and depression is similar between girls and boys, with the main difference observed being that the association was stronger in girls.

While body dissatisfaction appears to be a common denominator in adolescent depressive symptoms irrespective of sex/gender, underlying drivers of body dissatisfaction may differ between girls and boys as well as across other dimensions of identity. In other words, the relation of body dissatisfaction to pubertal development may be contingent on normative body ideals, which vary widely across numerous sociocultural constructions. Soares Filho et al. [82] surveyed over 2100 youth living in Brazil and found that girls were significantly more likely to report dissatisfaction related to feelings of being overweight, while boys were more likely to report dissatisfaction due to perceived thinness. These sexed/gendered experiences of body dissatisfaction may relate to depression and self‐hatred specifically [81] through the potential psychosocial consequences of meeting or not meeting “ideal” body standards that differ both by sex/gender and across pubertal development. This possibility is underscored in the present findings by a weak statistical trend towards an interaction between pubertal timing and sex/gender in predicting the likelihood of peripubertal body dissatisfaction, suggesting a tendency for sex/gender to influence how pubertal timing is associated with body dissatisfaction, while also suggesting important intracategorical variation. Indeed, research demonstrates that the relation between body dissatisfaction and pubertal timing varies considerably within sex/gender categories. An analysis of survey data from 218 girls living in United‐States found differential effects of early and late pubertal timing by ethnicity in relation to adolescent depressive symptoms, which were mediated by body esteem [83]. Understanding the relation between sex/gender, pubertal development, and body image has important implications for contextualizing the unique contributions of pubertal development to adolescent depression.

#### 4.1.3. Moderating Effects of Sex/Gender on Psychosocial Experiences Related to Depression

Two variables were found to predict adolescent depressive symptoms differently across sex/gender: family dysfunction at age 10–11 and postpubertal covert delinquency. Specifically, higher levels of family dysfunction predicted adolescent depressive symptoms only in boys, with no effect apparent for girls, while postpubertal covert delinquency predicted increased levels of depressive symptoms in girls only, with mean depressive symptoms being similar across boys irrespective of covert delinquency and girls who reported no covert delinquency (Figure 1b).

The effect of family dysfunction we observed in boys corroborates findings from sex‐segregated analyses [24]. In contrast, research conducted with children living in the USA, in predominantly Caucasian samples, suggests that family conflict in late childhood predicts adolescent depressive symptoms similarly in both girls and boys [84, 85]. These discrepancies could occur for any number of reasons, including regional or cultural variation, as well as cross‐study differences in the ways in which potentially diverse indices of family conflict are captured with available measures (e.g., caregiver‐reported vs. youth‐reported). Regardless of inconsistency in sex/gender differences, converging evidence suggests that childhood family dysfunction comprises a valuable source of context for understanding adolescent depressive symptoms and should be incorporated into biosocial models of adolescent depression. Previous work that integrates both parental and adolescent data regarding experiences of parent–child conflict during children’s pubertal development suggests important qualitative differences in parents’ vs. children’s attitudes about the timing of puberty as well as the nature of parent–child conflict during puberty according to children’s gender, and a low degree of overlap between parental and adolescent reports [86].

In line with Mendle et al. [35], we observed that earlier pubertal timing predicted increased reporting of covert delinquency postpubertally. In our mixed‐sex sample, this effect was observed irrespective of sex/gender. Past research suggests that early pubertal timing predicts adolescent delinquent behavior through increased sexual activity [87] and peer substance use [88, 89] in spare time activities, with no moderation by sex/gender where moderating effects were tested [87, 89]. Interestingly, sex/gender‐moderated factors other than pubertal timing in predicting the likelihood of reporting covert delinquency. Specifically, girls with experiences of childhood instability were significantly more likely to report covert delinquent behaviors than boys who had experienced instability, while no sex/gender effect was apparent for children with no experiences of instability (Figure 4).

However, despite the association between earlier pubertal timing and covert delinquency being unmoderated by sex/gender, the effect of participating in covert delinquent behavior on depressive symptoms in our study sample was only apparent in girls, suggesting variable pathways to adolescent depression across sex/gender. Investigation of sex/gender differences in the temporal dynamics of delinquent behaviors in relation to depression suggests that delinquency in girls predicts greater depressive symptoms, while in contrast, depressive symptoms tend to predict delinquent behaviors in boys [90, 91], although not consistently [92]. Our findings support the general idea that early pubertal development in girls favors a postpubertal depressogenic context (see also [35]).

### 4.2. Childhood Stressors and SES

Although childhood stressors were unassociated with variation in depressive symptoms, these experiences were associated with relevant peri and postpubertal psychosocial experiences relevant to adolescent depression. However, childhood stressors predicted variation in peripubertal body dissatisfaction and postpubertal covert delinquency. Specifically, experiences of loss and adversity/health stressors were both found to predict a lower likelihood of body dissatisfaction at age 13–14 irrespective of sex/gender. Further, as noted above, experiences of childhood instability interacted with sex/gender to predict covert delinquent behavior.

The absence of association between childhood stressors and pubertal timing in our sample could also be in part related to our operationalization of adversity domains and our study population. For instance, Shaul et al. [93] operationalized ‘threat’ as including experiences related to terrorism, living in a war zone, and sexual abuse, which were not accessible in our dataset. We binarized the occurrence of childhood stressors in three distinct dimensions to examine their relation to variation in pubertal timing [94] irrespective of the number of stressors experienced within a category. This prevented us from looking at the cumulative risk, which has been previously associated with psychopathology in childhood [95].

Lower childhood socioeconomic status predicted earlier pubertal timing but did not predict variation in early physical development (Supporting Information File 1). This finding aligns with other studies reporting that SES, as opposed to childhood stress exposure, corresponded with early pubertal timing [41, 96]. In contrast, Stenson et al. [45] investigated the association between early trauma and pubertal timing in African American youth from low‐income urban areas and observed that greater occurrence of early trauma corresponded with earlier pubertal timing in girls and delayed pubertal timing in boys. It is possible that effects of childhood stress exposure may be more apparent when investigation focuses on lower SES strata; constraining a study sample may provide higher resolution within a particular context.

### 4.3. Context‐Contingent Conceptualization of Sex/Gender in the Relationship Between Pubertal Development and Depression

A growing literature suggests that early pubertal timing constitutes a transdiagnostic risk factor for adolescent mental health, with inconsistent findings regarding the specificity of sex/gender effects [10, 11]. After integrating childhood, peri and postpubertal experiences into our model of adolescent depressive symptoms, effects of pubertal development were completely accounted for by psychosocial context, irrespective of our operationalization of puberty.

The present findings, together with previous work [12, 39, 50, 71], support taking a psychosocially contextualized approach to understanding whether and how variation in the timing of pubertal development may be associated with adolescent depressive symptoms. This approach places a greater theoretical focus on how experiences throughout pubertal development may vary according to socio‐structural context. For instance, Vijayakumar et al. [71] used factor analysis across 15 social environmental variables to quantify adolescents’ social “microsystem” (positive, negative, or exosystem [socio‐environmental]) in relation to pubertal timing and psychopathologies including depressive symptoms in girls and boys, and found interactions between pubertal timing and adolescents’ social microsystem that varied by sex/gender, microsystem, and diagnostic category (e.g., depressive vs. anxiety symptoms). Importantly, pubertal timing was only found to predict emotional and behavioral problems in adolescence in the context of an unfavorable social microsystem. Familial context has also been shown to moderate the link between early pubertal timing, cortico‐limbic connectivity, and adolescent depressive symptoms, with a positive family environment buffering against reduced cortico‐limbic functional connectivity associated with early pubertal timing [12].

A contextualized conceptualization of pubertal development has important implications to help arbitrate between and/or expand on theoretical models of the association between early puberty and adolescent psychopathology. Of several prominent theoretical models comprehensively reviewed in Ge and Natsuaki [97], the results of the present study and other recent work [71] best support the contextual amplification model and suggest future directions for expanding the context‐contingent conceptualization of pubertal development. Within this framework, sex/gender is considered as one of many sources of biosocial context informing the meaning that psychosocial experiences associated with pubertal development can have in relation to depression risk. In our work, sex/gender was not an independent predictor of any psychosocial experiences nor of adolescent depressive symptoms. Rather, sex/gender moderated some psychosocial experiences related to depression, but most effects were similar in boys and girls, suggesting that sex/gender might not be the most relevant category for understanding adolescent depressive symptoms within the study population. Growing literature demonstrates that pubertal development may be associated with different psychosocial experiences across ethnicity within the same sex/gender category [83]. The meaning that psychosocial experiences have varies across and within sex/gender, pointing to the importance of understanding the diversity of experiences that covary with pubertal development [98].

### 4.4. Analytical and Conceptual Considerations for Context‐Informed Analysis of Sex/Gender Across Pubertal Development

Currently in literature on depression and pubertal development, a common analytical strategy to assess effects of sex/gender is to conduct sex‐segregated analysis [99]. This approach limits a contextualized articulation by precluding adequate investigation of moderating effects of sex/gender [25–27] because the a priori segregation of boys and girls for analysis has the consequence of eliminating our statistical ability to reliably assess both differences and similarities. These limitations directly impact the nature and validity of inferences that can be made from our analyses [25]. Although there are relatively clear‐cut situations in which separating groups for disaggregated analysis is the most appropriate a priori strategy (e.g., studying oogenesis vs. spermatogenesis; [99]), most operationalizations of pubertal development in the current literature (Supporting Information File 1) do not refer to biological processes that differ fundamentally by sex/gender, but to broad differences in developmental timing, the potential impacts of which should not be assumed by default to differ according to sex assigned at birth [11]. It is also important to consider that, statistically, disaggregating groups for analysis is tantamount to presuming *a* priori that the identified groups reflect fundamentally different populations that cannot be directly compared.

An acontextual approach also tends to reinforce essentialist inferential leaps. When puberty is conceptualized and operationalized solely as a sex‐based biological phenomenon, effects tend to be interpreted as biologically essential [100, 101]. For example, the prevalence gap in depression by sex/gender [3] is often assumed to be related to biological differences that render girls more “vulnerable,” without demonstrating that such vulnerability exists beyond the observed prevalence gap. Experiences such as discrimination have been associated with differences in pubertal development as well as variation in circulating levels of gonadal hormones [102], reinforcing the biosocial nature of puberty and the importance of accounting for development experience in order to understand the role of puberty in adolescent mental health. Importantly, we do not suggest that hormonal or other physiological variation is irrelevant to understanding puberty in relation to adolescent depression. Rather, we promote a biosocial approach that incorporates exploration of the possibility that physiological change can be driven by variation in the type and frequency of psychosocial experiences to which early maturing youth are exposed. Gendered experiences themselves are context‐contingent [103].

## 5. Strengths and Limitations

The NLSCY is a longitudinal survey with information about children and youth acquired at their time of development, preventing retrospective bias. The use of clustered sampling maximized representative sampling. However, our analytical approach required data on a number of factors acquired across several data collection cycles, leading to the exclusion of participants due to missing data. Our sensitivity analyses suggested that data were missing not at random, limiting the validity of data imputation techniques [104]. As such, we did not impute missing data, and our final analytic sample is not nationally representative, which may limit generalizability. That said, our aim is not to produce results that can be understood in a decontextualized (i.e., generalizable) manner. Instead, we attempt to illustrate the importance of accounting for psychosocial variables that vary across populations and settings. This contextualized approach promotes better understanding of the complexity of sex/gender, puberty, and depression as biosocial constructs.

In the present sample, over 95% of participants reported Caucasian ethnicity, and participants self‐reported sex as female or male (i.e., no representation of sex/gender diversity beyond binary sex). Lack of diversity in research samples in studies of pubertal development has also been highlighted as a more general issue in this literature [15]. Increasing diversity and representation in research on sex/gender, puberty, and depression continues to be an important priority for future work, as differential effects of pubertal timing by race/ethnicity have been documented [83]. Considering the context‐contingent diversity of experiences is central.

Although the NLSCY is one of the most comprehensive longitudinal surveys of its kind, it does not include data on key gendered variables (e.g., sexual violence), which are known to be disproportionally experienced by girls and associated with both early pubertal timing and depression in girls [70, 105, 106]. Similarly, the available data did not support investigating variation in the cumulative severity or participants’ experiences of early‐life stressors.

We tested two operationalizations of early pubertal development: pubertal timing and early physical pubertal development, defined as having an advanced pubertal status at or prior to age 11. Both constructs were derived from children’s self‐reported pubertal status on the PDS rather than assessed by an experimenter. This approach is commonly employed but subject to reporting bias [107, 108]. Further, our designation of early puberty based on physical development at or prior to age 11 may not adequately capture early pubertal development in boys, due to the well‐documented finding that girls tend to begin puberty earlier than boys [15]. It may be valuable in future work to consider designating early puberty at sex/gender‐contingent age ranges to better capture experiences of early physical development in boys; this issue may also help to address some of the inconsistency in the literature regarding sex/gender differences related to early pubertal development defined with respect to age.

Our findings regarding standardized puberty scores do not seem to suggest any critical sex/gender differences in pubertal timing relative to same‐age peers. However, the impacts of early physical development may benefit from further refinement considering potential gendered differences in experiences of body dissatisfaction during puberty. Other relevant operationalizations of pubertal development such as pubertal tempo, have been shown to be associated with mental health outcomes and should be further explored. Finally, refinement in the assessment of pubertal development with the inclusion of nocturnal emission and changes in gonadal hormones will expand the contextual landscape of puberty.

The present analyses focused on factors predicting depressive symptoms rather than the likelihood of meeting clinically relevant cut‐offs for diagnosis of probable depression (CES‐D ≥ 12). This analytical choice was made to align with previous research focusing on symptoms, much of which deals with sex/gender‐related differences in subthreshold levels of depressive symptoms. It should be noted that factors predicting depressive symptom levels may differ from those predicting diagnosis. Moreover, the functional interpretation of a significant difference in sub‐threshold depressive symptoms relates to depression risk through the risk of symptoms escalating to diagnostic levels, which may occur via numerous pathways. Notably, in our dataset, we observed age‐related differences in the sex/gender distribution of CES‐D mean scores vs. the percentage of participants reaching the cut‐off score of 12. In adolescents captured at age 16–17, CES‐D mean scores differed significantly by sex/gender, but the prevalence of probable depression did not, while in adults we observed the opposite pattern (Table 1). Subthreshold depressive symptoms have been shown to be predictive of the onset of major depression within 2 years [109], but additional person‐level risk and protective factors may help to account for this escalation [110]. Some researchers argue that adopting a more dimensional, spectrum‐based model of depressive symptoms is important for informing our understanding of risk [111]. However, focused longitudinal work on trajectories of symptom change across development and into adulthood, following research designs such as that of Conley and Rudolph [68, 112] will be valuable for informing this question.

Finally, we note that the present study contains a large number of statistical comparisons. Given the exploratory nature of our analysis of interactions, we did not adjust our critical *p*‐value from the common standard of *p* < 0.05. We note, however, that a majority of findings reported remain significant at a more conservative threshold of *p* < 0.01, and underscore that our analysis is intended to demonstrate the importance of accounting for psychosocial context in attempting to explain the link between pubertal development and depression, rather than to advance strong conclusions regarding the most influential risk factors for adolescent depressive symptoms. Further work is necessary to identify the most influential psychosocial risk factors covarying with the timing of pubertal development across sex/gender.

## 6. Conclusions and Future Directions

Our findings contribute to highlighting the context‐contingent nature of pubertal development and sex/gender as predictors of depressive symptoms in late adolescence. We find no evidence that pubertal development is a key contributor to sex/gender‐related differences in rates of adolescent depressive symptoms. Moreover, we find no evidence of unqualified sex/gender differences in depressive symptoms after accounting for a host of childhood, peri and postpubertal psychosocial moderators identified in previous work. This finding calls for broader contextualization of the globally documented sex/gender gap in depression prevalence, which has resulted in essentialist hypotheses about women’s biological vulnerability to psychopathology [113]. Sex/gender was a significant moderator of some, but not all, psychosocial experiences that were important for understanding variation in depressive symptoms. Research attempting to understand pubertal development and sex/gender in relation to health must pursue understanding of individual differences in risk across multilevel individual, psychosocial, and sociostructural contexts. Importantly, because much research on putative sex/gender differences in the association between pubertal development and depressive symptoms overlooks these psychosocial experiences, the current findings add to a growing literature that strongly supports an operationalization of pubertal development as a context‐contingent risk factor for adolescent depressive symptoms. This integration is a critical and necessary step to advance more comprehensive theories of pubertal development that mitigate essentialism and the potential for inadvertently pathologizing puberty in girls.

## Funding

The current study was supported by an internal UNBC Research Funding Award (Grant 6007502) to Caroline Sanders.

## Ethics Statement

Research Ethics Board approval was not required for the analysis of extant Statistics Canada data. All Statistics Canada policies regarding data access and participant confidentiality were followed.

## Conflicts of Interest

The authors declare no conflicts of interest.

## Endnotes

1

## Supporting Information

Additional supporting information can be found online in the Supporting Information section.

## Supporting information


**Supporting Information** The NLSCY is a large, complex longitudinal dataset from which information been merged and recoded across multiple cycles to facilitate the present cross‐sectional analysis. Supporting material for the present study includes detailed notes on how the survey data were handled, including variable coding, handling of missing data, and information on weighting strategies (Supporting File 1); regression tables for all models reported (Supporting File 2); and comprehensive demographic descriptive summary statistics regarding attrition rates (Supporting Table 1) and participant exclusions resulting from missing data (Supporting File 2). These materials are provided for transparency and replication. Box 1 definitions are provided to illustrate the diversity of operationalizations of puberty as a research construct.

## Data Availability

The National Longitudinal Survey of Children and Youth is a Statistics Canada dataset available to university‐based researchers via application through the Canadian Research Data Centre Network. Data used for analysis in the present study are not shareable due to Statistics Canada confidentiality rules.

## Footnotes

1
^1^We use the term sex/gender here to reflect the entanglement of these constructs [114], or in other words, to reflect that gender and sex are interdependent and therefore inadvisable to measure and/or study in isolation. Importantly, while some scholars advocate the use of the term gender/sex rather than sex/gender, we use sex/gender here to reflect the operationalization of “sex” in the present dataset and in the literature more broadly, but note that for our purposes, sex/gender and gender/sex are considered functionally equivalent terminologies.
