# Supplementary material for: Puberty in Context: Accounting for Psychosocial Experiences in the Association Between Pubertal Timing, Sex/Gender, and Adolescent Depressive Symptoms in Canadian Youth
Source: Depress Anxiety. 2026 Apr 12;2026:5568871. doi: 10.1155/da/5568871 (PMC13071339; doi:10.1155/da/5568871)
Supplement: Supplementary file 1 — Supporting Information The NLSCY is a large, complex longitudinal dataset from which information been merged and recoded across multiple cycles to facilitate the present cross‐sectional analysis. Supporting material for the present study includes detailed notes on how the survey data were handled, including variable coding, handling of missing data, and information on weighting strategies (Supporting File 1); regression tables for all models reported (Supporting File 2); and comprehensive demographic descriptive summary statistics regarding attrition rates (Supporting Table 1) and participant exclusions resulting from missing data (Supporting File 2). These materials are provided for transparency and replication. Box 1 definitions are provided to illustrate the diversity of operationalizations of puberty as a research construct. [file DA-2026-5568871-s001.zip › Supplementary File 1 - Additional notes on data coding.docx]

**Supplemental File 1**

**Operationalizations of puberty**

| *Pubertal development*  A complex biopsychosocial process involving encompassing hormonal (e.g., activation of the HPG axis), morphological (e.g., development of secondary sexual characteristics), physiological (e.g., menarche, nocturnal emission), and psychosocial and emotional changes (e.g., changes to peer groups). | |
| --- | --- |
| *Pubertal status*  Reflected in measures of physical developmental milestones, including morphological changes such as the development of secondary sex characteristics (e.g., Tanner stages), and/or physiological changes such as adrenarche. | |
| *Timing of pubertal development*  Any measure which considers pubertal development in relation to the timing of pubertal development milestones. Measures related to timing of pubertal development can be broadly characterized according to whether the timing of pubertal development is considered in relation to age, or in a manner relative to the timing of development in peers. | |
|  | ***Early pubertal development*** ***(objective):*** Early pubertal development can be objectively operationalized through combining a measure of pubertal status with age. As Vijayakumar & Whittle (2023) note, this can be achieved through collecting age-restricted participant samples or by controlling for age in analyses of pubertal status. It is also possible to operationalize early pubertal development by referencing normative data on mean age at specific pubertal status milestones (e.g., mean age at menarche). |
|  | ***Pubertal timing (peer-relative):*** Pubertal timing is a peer-relative measure that uses scores on measures of pubertal status (e.g., the Pubertal Development Scale; Petersen et al., 1988) to create age- and sex-standardized pubertal timing scores, where a score of 0 reflects pubertal timing on-par with that of same-age, same-sex peers, while positive and negative scores reflect earlier or later peer-relative pubertal development, respectively. |
|  | ***Pubertal tempo (peer-relative):*** The pace of pubertal development relative to same-age, same-sex peers. |
|  | ***Pubertal synchrony (peer-relative):*** The occurrence of specific pubertal development milestones (e.g., development of body hair) relative to same-age, same-sex peers. |

**Further notes on data coding from the NLSCY**

**Attrition**

We conducted preliminary demographic comparisons of participants who completed cycle 8 measures with those lost to attrition (Supplementary Table 1). Biases in survey completion were apparent. Specifically, participants who did not complete all cycles of data collection reported a higher number of household members, generally lower levels of PMK education, and were significantly less likely to have been born in Canada. Further, participants lost to attrition reported significantly lower cycle 1 SES, slightly fewer experiences of childhood loss, greater frequency of experiences of childhood instability, and significantly higher mean scores on the child conduct problems scale at cycle 1.

**Participant exclusions**

Biases in survey completion were also apparent while examining missing data to conduct exclusions (Supplementary Table 2). Males were significantly more likely to be excluded from the analytic sample due to missing data. Cycle 1 SES was significantly lower in excluded participants, and PMKs of excluded participants were less likely to report post-secondary education. Excluded participants were also significantly more likely to report early pubertal development (≤ age 11), more likely to report experiences of childhood instability, and more likely to report participation in overt delinquent behaviour, while included participants were significantly more likely to report covert delinquency. Scores for PMK depression, conduct problems, and family dysfunction were also significantly higher in the excluded sample.

**Weighting strategies for analysis**

The NLSCY includes multiple sets of survey weights including cross-sectional weights computed for each cycle as well as longitudinal weights updated at each cycle of data collection. All survey weights are provided by Statistics Canada as part of the NLSCY dataset and all types of weights provided account for the complex non-random clustered sampling procedures used by Statistics Canada to ensure a nationally representative sample. Longitudinal non-funnel and funnel weights additionally account for the changing demographics of the Canadian population over time, while longitudinal non-funnel weights further account for participant attrition. The present study merged longitudinally collected data from multiple cycles to conduct a cross-sectional analysis using variables captured at different age ranges. However, it is not possible to ‘merge’ survey weights across cycles in the same way, so a single set of weights must be selected for each analysis. Our strategy for weighting participants’ data in the present analysis was guided by consultation with Statistics Canada data analysts familiar with the weighting strategies used for the NLSCY.

The sample of interest in the present study comprised youth aged 8 to 11 years old at cycle 1 with complete data on depressive symptoms at cycle 8 (age 21-25) to allow for the potential exploration of depressive symptoms in both adolescence and adulthood. To compare demographic characteristics at cycle 1 for those who completed all cycles of data collection to those of participants lost to attrition, we used cycle 1 cross-sectional survey weights. Cycle 1 was chosen as a basis for comparing these demographics largely because participants lost to attrition had incomplete data at later cycles. All other analyses focused on the sub-sample of NLSCY participants aged 8-11 at cycle 1 who also completed cycle 8 measures (i.e., participants who completed the longitudinal survey). For these analyses, we used Cycle 8 longitudinal funnel weights to account for attrition and population change over time.

**Missing data & creation of new variables**

At each stage of variable coding, a series of interim variables was created to maintain a clear record of missing data on every item of interest at every cycle. When items were subsequently combined into composite measures (e.g., combining information about pubertal status with participant experiences to code post-pubertal variables), when data were merged across two or more cycles into a single variable (e.g., data captured at age 14-15 involved merging data from cycles 4 and 5, as half the sample fell into this age range at each of these cycles), or when variables had to be coded separately by group and then merged into a single variable (e.g., coding girls and boys on pubertal status and timing), the new variable would retain an empty cell wherever data could not be meaningfully combined due to missing values.

All newly created variables were cross-checked with original and interim variables to ensure reliable coding throughout. Although this would seem a rather straightforward necessity in data coding for any study, is worth specially noting here as the NLSCY is a large and complex dataset with numerous codes for different types of missing data (e.g., “valid skip”, “not stated”, “don’t know”). For instance, a “valid skip” was coded whenever a participant fell out of scope for a questionnaire item, and it was important for our data coding and participant exclusion procedures to distinguish between “valid skip” (i.e., participants who were not asked a particular item) from truly missing data (i.e., participant was asked but did not provide a response). Two significant examples of this included 1) the assessment of puberty at Cycle 1, wherein only 10-11 year-olds were asked about their pubertal development, resulting in “missing data” for all children aged 8-9 at Cycle 1, and 2) in dealing with nested questions wherein a participant was only asked certain items based on responding “Yes” to a previous item. We found that creating new variables that combined information from multiple questionnaire items required substantial attention to the issue of how missing data were coded within and across cycles.

Though all participants in the final analytic sample completed cycle 8 measures, some of these participants did not complete an interim cycle (e.g., complete data for all cycles except cycle 4). We chose to include participants with only partial data on items comprising some of our composite measures (e.g., post-pubertal delinquency measures), provided that most key variables of interest (e.g., transition from pre- to post-pubertal) were adequately captured from available data. We chose this strategy rather than excluding these participants for three reasons. First, the NLSCY questionnaire itself is somewhat inconsistent across cycles, as the survey was iteratively re-evaluated during the longitudinal frame, resulting in the revision, removal and/or addition of items in later cycles. Second, and for the same reason, some items were only available at later cycles. Similarly, across all cycles, some items were only asked of children falling into certain age ranges (e.g., particularly for delinquent behaviour items or about children’s dating and sexual experiences). Third, for variables where missing data from an interim cycle were an issue (mostly post-pubertal variables) we were interested only in capturing *any* occurrence of a particular event (i.e., *any* post-pubertal delinquency), and as such we expected that partial missing data would be reasonably unimpactful. It is possible that we may have missed these pos-pubertal experiences in some participants who did not respond at a particular cycle, or coded them as “first occurred post-pubertally” if a pre-pubertal experience was missed, but we expected this issue to be minimal. Of participants who completed cycle 8 measures, we examined the final set of variables coded for analysis for missing data and excluded all participants with missing data on coded variables of interest.

**Sex/gender diversity in the sample**

Sex/gender was collected in early cycles by PMK report, and in cycle 8 by participant self-report. We evaluated correspondence between these measures to assess sex/gender diversity in the sample. A small number of participants^^[[1]](#footnote-1)^^ self-reported a gender that differed from cycle 1 PMK report. We did not explicitly exclude these participants, though it is worth noting that they were not included in the final analytic sample due to missing data on other measures unrelated to sex/gender, thus our final sample includes only self-identified females and males.

**Issues encountered with measurements across cycles**

Due to our focus on coding the occurrence of post-pubertal life events, which required variable coding at each cycle to link pre- or post-pubertal status with the occurrence of each event, we noted that a small number of participants reported non-linear pubertal status (e.g., endorsed PDS items resulting in an assignment of post-pubertal status at cycle 2, but pre-pubertal status at cycle 3). These participants were also excluded from the analyses due to possible unreliability of reporting.

Similarly, due to the requirement to code numerous variables relative to participant age (e.g., pubertal development and associated peri- or post-pubertal variables), we noted that some participants were captured twice on a small number of measures intended to be administered to children falling into a certain age range. The NLSCY codes multiple variables capturing participant age including initial cohort assignment (e.g., 8 years old at cycle 1) and actual age at time of interview in subsequent cycles (e.g., a child who should be 10 years old at cycle 2 according to cycle 1 cohort assignment may have been captured at age 9 or 11 depending on the time of year of survey completion). Moreover, questionnaire items or scales intended to capture children at specific ages were sometimes administered according to cohort assignment age, while other such measures were administered according to actual age at time of interview. Therefore, some participants were administered the same age-specific measure at two cycles. In these cases, we used the earliest available measure based on assigned cohort age at cycle 1.

**Additional details on measures**

All measures are detailed more extensively in the Statistics Canada user guide for cycle 1 of the NLSCY. Below is a brief overview.

**Socio-economic status (SES).** SES was indexed using a single variable combining five dimensions of household SES including highest education level of the PMK and spouse/partner, occupational prestige of the PMK and spouse/partner, and household income. All five dimensions were standardized and averaged to produce an overall SES score variable (Statistics Canada, n.d.).

**Conduct problems.** Conduct problems were indexed via scores on the NLSCY conduct problems and physically aggressive behaviour scale, with items drawn or adapted from the Ontario Child Health Study. Items included ratings of children’s propensity to misbehave or act aggressively and were reported by the PMK. Higher scores indicate more conduct problems.

**Emotional problems.** Emotional problems were indexed via total scores on the NLSCY emotional disorder - anxiety scale score, with items drawn or adapted from the Ontario Child Health Study. Items included ratings of children’s overall happiness and sadness, anxiety, and worry, and were reported by the PMK. Higher scores indicate a higher level of emotional problems.

**Family dysfunction.** Overall functioning of the family system was assessed with a widely used scale developed at McMaster University and indexing issues with resolving misunderstandings, avoidance or expression of feelings in the family, feeling accepted, and easy of family decision-making, among others, reported by the PMK. Higher scores indicate more dysfunction in the family system.

**Perceived parental rejection.** Perceived parental rejection was self-reported by participants across several items comprising a total scale developed by Lempers, Clark-Lempers, & Simons (1989). This scale was also used in the Western Australia Child Health Survey. Higher scores indicate more perceived parental rejection.

**Delinquent behaviour.** We identified items capturing delinquent behaviour based on previous work on the association between pubertal development and delinquency. Several modes of operationalizing delinquent behaviours into categories have been proposed, including Haynie’s (2003) 3-level framework separating “party deviance” from minor and serious delinquency; Loeber’s (1996) binary framework of covert and overt delinquency; and Achenbach’s (2001) framework in developing the Youth Self-Report measure of aggressive and non-aggressive delinquency. We opted for a binary framework generally corresponding to the distinctions of Loeber (1996) and Achenbach (2001), considering covert/non-aggressive delinquency and overt/aggressive delinquency. Items and the categories into which they were coded are summarized in a table below.

Each item was coded as occurred/not occurred for every participant at each of cycles 1-5, after which the timing of each occurrence relative to pubertal development was coded to produce interim variables indexing whether the first recorded occurrence of each item was post-pubertal (first occurrence post-pubertal=1) or not (not occurred or first occurrence pre-pubertal=0). Items were then combined into composite measures indexing the occurrence of post-pubertal covert and overt delinquency (1=any first occurrence of post-pubertal delinquency; 0=no occurrence or first occurrence pre-pubertal).

Notably, many of these items were not asked of younger children, and some were only introduced to the NLSCY at later cycles, or for a limited number of cycles (e.g., the item indexing gang membership was only asked at cycles 3 and 4). In addition, due to the aforementioned issue of missing data for some participants for a whole survey cycle, the data on delinquent behaviour in the present study are somewhat incomplete, and intended in the present analysis to serve as an important contextualizing factor that should be further studied in tandem with sex/gender and the timing of pubertal development.

| **Questionnaire item content** | **Delinquency category code** |
| --- | --- |
| Questioned by police | Covert/non-aggressive |
| Part of a gang that breaks the law | Overt/aggressive |
| Destroys things belonging to others | Overt/aggressive |
| Fought someone who required medical attention | Overt/aggressive |
| Attacked someone with the intent to harm | Overt/aggressive |
| Sold drugs | Covert/non-aggressive |
| Touch someone sexually knowing they might object | Overt/aggressive |
| Vandalize property | Covert/non-aggressive |
| Steal from a store or school | Covert/non-aggressive |
| Take money from parents without permission | Covert/non-aggressive |
| Break and enter with the intent to steal | Overt/aggressive |
| Threaten another person for personal gain | Overt/aggressive |
| Try to force sexual contact | Overt/aggressive |
| Stealing (large items such as a car) | Overt/aggressive |
| Setting fires | Overt/aggressive |

References

Achenbach, T. M., & Rescorla, L. A. (2001). Manual for the ASEBA School-Age Forms & Profiles. In: Burlington, VT: University of Vermont, Research Center for Children, Youth, & Families.

Haynie, D. L. (2003). Contexts of risk? Explaining the link between girls' pubertal development and their delinquency involvement. In (pp. 355-397): University of North Carolina Press.

Lempers, J. D., Clark-Lempers, D., & Simons, R. L. (1989). Economic hardship, parenting, and distress in adolescence. *Child Dev*, *60*(1), 25-39. <https://doi.org/10.1111/j.1467-8624.1989.tb02692.x>

Loeber, R. (1996). Developmental continuity, change, and pathways in male juvenile problem behaviors and delinquency. In *Delinquency and crime: Current theories.*

1. N is not reportable due to Statistics Canada confidentiality rules [↑](#footnote-ref-1)
